# Benchmarking pangenome dynamics and horizontal gene transfer in *Mycobacterium marinum* evolution

**DOI:** 10.3389/fmicb.2025.1537826

**Published:** 2025-06-17

**Authors:** Khandker Shahed, Sk Injamamul Islam, Papungkorn Sangsawad, Won-Kyo Jung, Patima Permpoonpattana, Nguyen Vu Linh

**Affiliations:** ^1^BioMac Lab, Dhaka, Bangladesh; ^2^School of Animal Technology and Innovation, Institute of Agricultural Technology, Suranaree University of Technology, Nakhon Ratchasima, Thailand; ^3^Department of Agricultural Science and Technology, Faculty of Innovative Agriculture, Fisheries and Food, Prince of Songkla University, Surat Thani Campus, Surat Thani, Thailand; ^4^Department of Animal and Aquatic Sciences, Faculty of Agriculture, Chiang Mai University, Chiang Mai, Thailand

**Keywords:** benchmark, pathogens, genome, alternatives to antibiotic, SNPs

## Abstract

Horizontal gene transfer (HGT) is a key driver of microbial evolution, promoting genetic diversity and contributing to the emergence of antibiotic resistance. This study explores the pangenome dynamics and HGT in *Mycobacterium marinum* (*M. marinum*), a close relative of *Mycobacterium tuberculosis*. Multiple pangenome datasets were analyzed to quantify gene gain, loss, and pangenome openness, utilizing Panstripe and a Generalized Linear Model (GLM) framework to assess gene presence/absence across strains. Additionally, a comparative benchmarking analysis of gene ontology (GO) annotations were conducted using eggNOG and InterProScan to evaluate their functional annotation accuracy. Our findings demonstrated significant differences in gene gain and loss rates, suggesting variations in annotation accuracy and the presence of mobile genetic elements (MGE). Single nucleotide polymorphisms (SNPs) were also identified, highlighting the genetic variability that may impact strain-specific traits such as pathogenicity and antibiotic resistance. Pangenome of *M. marinum* was characterized as highly open, with substantial variability in gene content, reflecting ongoing genetic exchange and adaptability. Functional annotation benchmarking demonstrated that eggNOG and InterProScan provided complementary insights, with each tool excelling in distinct strengths of gene function identification. Overall, these findings highlight the complex interplay between HGT, pangenome evolution, and antibiotic resistance in *M. marinum*, and the analytical framework presented here provides a robust approach for future studies aiming to inform therapeutic interventions and vaccine development.

## 1 Introduction

*Mycobacterium marinum* causes tuberculosis-like disease in poikilothermic animals and acts as an opportunistic pathogen in humans, typically manifesting as localized skin infections due to its temperature-dependent growth restriction (Ramakrishnan and Falkow, 1994). Unlike *Mycobacterium tuberculosis*, which has adapted specifically to humans, *M. marinum* retains a generalist ecological strategy, infecting diverse animal hosts and protozoa. Despite its larger genome, *M. marinum* shares close phylogenetic ties with *M. tuberculosis*, and exhibits striking similarities in cellular infection mechanisms (Cardenal-Muñoz et al., 2018; Stinear et al., 2008). Originally misclassified as *M. piscium* due to its association with marine fish, *M. marinum* is now recognized as a globally distributed pathogen (Aubry et al., 2017). It thrives in diverse aquatic environment—saltwater, brackish systems, and both stagnant and flowing freshwater—infecting over 150 fish, frog, eel and oyster species (Aronson, 1926; Beecham et al., 1991). Human infections are generally cutaneous, though immunocompromised individuals may experience deeper tissue invasion or systemic spread (Tsiolakkis et al., 2023). Scientific interest in *M. marinum* stems from its genetic similarity to *M. tuberculosis* and its utility in modeling tuberculosis pathogenesis using goldfish (*Carassius auratus*) (Ruley et al., 2004). Recent attention has intensified due to parallels with *M. ulcerans* infections and the growing popularity of aquarium-related activities. which may elevate clinical case rates (Aubry et al., 2017).

Pathogenetically, *M. marinum* and *M. ulcerans* from a distinct pathogenic clade within non-tuberculous mycobacteria (NTM) (Wolinsky, 1992), sharing > 98% nucleotide identity with *M. ulcerans* in key biomarkers, including *16S rRNA*, *rpoB*, *gyrA/B*, and *hsp65* (Dauendorffer et al., 2003; Stinear et al., 2000; Stinear et al., 2008). Early genomic comparisons of *M. marinum* strains (MB2, Europe, and the human-derived M strain) suggested highly similarity (Kurokawa et al., 2013), but expanded analyses of 15 human- and fish-derived isolates revealed unexpected genomic diversity (Das et al., 2018). Divergence primarily affects lipid metabolism, virulence factors, stress response pathways, underscoring the need for complete genome-based comparisons to resolve strain-specific adaptations (Vasudevan et al., 2020). DNA-DNA hybridization and mycolic acid profiling further confirm *M. marinum* and *M. ulcerans* as the closest relatives of *M. tuberculosis* (Rogall et al., 1990; Tønjum et al., 1998).

Advances in whole-genome sequencing have revolutionized bacterial phylogenetics, enabling precise strain typing, antibiotic resistance profiling, and outbreak surveillance (Abdella et al., 2023; Jin et al., 2020; Pérez-Pascual et al., 2017). While prior studies explored *M. marinum*’s genetic relationships (Das et al., 2018; Kurokawa et al., 2013), critical gaps remain in linking genomic diversity to atypical virulence factors and antimicrobial resistance (AMR). The growing availability of complete genomes now permits robust pangenome analyses, which are essential for distinguishing core and accessory genes, identifying SNPs/indels, and resolving taxonomic ambiguities (Reis and Cunha, 2021). Such approaches also address the limitations of traditional biomarkers, which often fail to capture taxonomy relationships across bacterial serotypes.

Horizontal gene transfer (HTG)—a primary driver of virulence and AMR genes acquisition—further complicates *M. marinum*’s genomic landscape (Mahmoud et al., 2022). To clarify these dynamics, a comparative genomics on eight complete genomes of *M. marinum* was performed. Subsequently, the pangenome and core genome were defined, and the average nucleotide identity (ANI) was calculated. The pangenome gain and loss rate was also analyzed. Collectively, this study would provide insights into the genomic features of the species and reveal highly conserved virulence genes along with their associations with antimicrobial drug resistance.

## 2 Materials and methods

### 2.1 *Mycobacterium marinum* dataset selection and quality assessment

A total of 100 distinct *M. marinum* genome sequences (including contigs, scaffolds, chromosomes, and complete sequences) are currently available in the NCBI genome database. On September 12, 2024, 8 complete genomes of *M. marinum* were downloaded. To further refine the data, the CheckM program (version 1.2.1) (Parks et al., 2022) was employed for genome quality filtration. The criteria for genome completeness were set at a minimum of 90%, while the maximum allowed contamination was 5%. Recent studies have emphasized the importance of selecting a high-quality and appropriate dataset for pangenome research (Wu et al., 2021; Wu et al., 2022). This careful selection ensures the reliability of downstream analyses, leading to more robust insights into pangenome and potential SNPs within the species. Supplementary Table 1 presents genome assembly, accession numbers, and genomic information, including genome size, gene count, and protein-coding genes. Only whole genomes were analyzed to ensure the most accurate representation of strain virulence genes and potential identifying biomarkers.

### 2.2 Average nucleotide identity

To verify species relationships, we performed nucleotide-level comparisons of all potential genome combinations using the ANI approach. The Python script PyANI v0.2.722 was used to compute pairwise ANI values using two different methods, the MUMmer (Kurtz et al., 2004) and the BLAST + method (Camacho et al., 2009) employing the ANIm and ANIb options, respectively. All strains classified as *M. marinum* were chosen for further analysis, while those misidentified were discarded. This decision was based on setting a threshold value indicating that the ANI of the same species exceeds 94% (Goris et al., 2007; Richter and Rosselló-Móra, 2009).

### 2.3 Building pangenome

To maintain consistency and standardization, the genomes of the *M. marinum* strains examined were re-annotated for their functional attributes using the Prokka suite version 1.14.6 (Podrzaj et al., 2022). The software Roary (Page et al., 2015) and Panaroo (Tonkin-Hill et al., 2020) with a default identity threshold of 95%, was employed to construct the core and pan-genome of *M. marinum*. Briefly, genes have been categorized into four distinct groups: core genes, present in more than 99% of genomes; softcore genes, found in most strains, specifically in over 95% but fewer than 99%; shell genes, identified in 15% to less than 95% of the strains; and cloud genes, which are observed in fewer than 15% of the total strains. In addition, the pangenome was analyzed using Heap’s Law (Rajput et al., 2023) and Power Law fit (Tettelin et al., 2005) in conjunction with the custom script^1^ and the “ggcaller v1.3.0” in Python programing (Horsfield et al., 2023), respectively. This approach allowed for the calculation of constant variables and the application of the least squares method to fit an exponential regression decay model to both the core genome and singletons. Heap’s Law was used to determine the fixed parameters from the pangenome with the formula n = κN^γ^, where n represents the number of pangenome genes and N is the number of genomes, least-squares fit is represented by the equation n = k × exp [-x/t] + tgθ, where n is the number of genes, and k, t, and tgθ are independent variables (Islam et al., 2025; Mwamburi et al., 2024). This algorithm is utilized to ascertain the number of genes that will compose the core genome upon stabilization (Soares et al., 2013) and to offer an approximate calculation of the genes contributed by each freshly sequenced genome. Additionally, the complete pangenome for the entire dataset of genomes was generated using anvi’o v8 (Eren et al., 2015), followed by the pangenomics procedure described in this guide: https://merenlab.org/2016/11/08/pangenomics-v2/2/. In brief, the following scripts were executed: anvi-gen-contigs-database, utilized to create a database, with Prodigal v2.6.3 (Hyatt et al., 2010) for identifying open reading frames in contigs, and anvi-run-ncbi-cogs, which provided gene annotations by utilizing NCBI Clusters of Orthologous Groups database (Tatusov et al., 2000). The genome database was created using anvi-gen-genomes-storage and anvil-pan-genome for visualization. Anvi’o employs the DIAMOND tool to compute the similarity between each amino acid sequence in every genome and all other amino acid sequences across all genomes in the dataset and then uses the MCL algorithm (van Dongen and Abreu-Goodger, 2012) to detect clusters in the results of amino acid sequence similarity.

### 2.4 Genome synteny and SNPs identification

Gene synteny analysis was performed using the Mauve program version snapshot_2015_02_13 (Edwards and Holt, 2013) and its progressive Mauve algorithm to detect potential gene rearrangement events. The software segmented the genomes into predefined fragments and used this information to perform numerous genome alignments. These alignments identified local collinear blocks (LCB) which were then visualized in a figure. This figure serves as a tool to identify and analyze rearrangements within the genomes. Subsequently, a customized Python script called msa2snp.py^2^ was employed to detect all SNPs within the target genes. The results of SNP identification were manually curated to identify the most informative SNP, found exclusively in all *M. marinum* complete genomes.

### 2.5 Functional annotation and identification of gene encoders

BLAST searches were performed for all protein-coding genes in the National Centre for Biotechnology Information (NCBI) database, which was accessed at https://www.ncbi.nlm.nih.gov/on 20.09.2024. The entire genome, including repetitive elements, was annotated using eggNOG-mapper v2 (Cantalapiedra et al., 2021) and InterProScan (Quevillon et al., 2005) in a Linux environment. The sequence alignment process entailed mapping each sequence with either the hidden Markov model (HMM) or DIAMOND to match it with the eggNOG database. The ideal matching sequence of the target sequence is classified according to its taxonomy and further categorized and annotated utilizing gene ontology (GO) (Gene Ontology Consortium, 2015) and Kyoto Encyclopedia of Genes and Genomes (KEGG) pathways (Kanehisa et al., 2017) through the application of the clusterProfiler v.3.19 package in R (Xu et al., 2024). Furthermore, BLAST analysis was performed to examine the 80 genomes for the presence of genes linked to virulence and pathogenicity. We employed BLASTN via ABRicate v1.0.1^3^ (de Man and Limbago, 2016) and the Virulence Factor Database (VFDB) (Liu et al., 2022) to examine the occurrence of virulence factors and antibiotic resistance gene in all strains of *M. marinum*. The analysis was performed using a 90% identity threshold and a 30% coverage threshold.

### 2.6 Region of genomic plasticity

To investigate the RGP in eight complete genomes of *M. marinum*, PPanGGOLiN software^4^ (Gautreau et al., 2020) was utilized with “panrgp” command. Genomic plasticity regions, which include genomic islands, prophages, and other variable elements, were identified using PPanGGOLiN’s comprehensive pangenome analysis capabilities. Initially, the genomes were prepared and formatted according to requirements based on the sample input dataset of the software. PPanGGOLiN was then employed to perform a detailed analysis, identifying regions of genomic variability and plasticity across the *M. marinum* strains. The tool provided insights into the distribution and composition of these plastic regions, allowing to map and characterize their presence and variability among the genomes. This analysis revealed the dynamic regions contributing to the genomic diversity and adaptability of *M. marinum*.

### 2.7 Pangenome gain and loss rate analysis

Horizontal gene transfer is a vital factor in the evolution and diversification of numerous microbial species. The ensuing dynamics of gene acquisition and loss can significantly impact the emergence of antibiotic resistance and influence the development of vaccines and therapeutic interventions (Tonkin-Hill et al., 2023). Panstripe^5^ improves the post-processing of bacterial pangenome analyses (Tonkin-Hill et al., 2023). Panstripe accepts a phylogeny and a gene presence/absence matrix in Rtab format, which can be generated by tools like Panaroo and Roary. Utilizing a Generalized Linear Model (GLM) framework allows for straightforward comparison of slope terms across different datasets. The “compare_pangenomes” function analyzes the interaction term between the datasets alongside the core, tip, and depth terms. A significant *p*-value for the tip term indicates that the two pangenomes differ in the rates of gene presence and absence observed at the tips of the phylogeny. This variation is often attributed to differences in annotation error rates between datasets or to fluctuations in the gain and loss of highly mobile genetic elements that may not persist long enough to be detected across multiple genomes. A significant *p*-value for the core term suggests differing rates of gene gain and loss between the two datasets. Although the depth term is less critical, it reflects discrepancies in the ability to detect older gene exchange events between the two pangenomes. The dispersion parameter reveals whether there is a notable difference in the dispersion of the two pangenomes, implying that the relationship between gene exchange rates and the size of each event varies between the two datasets. The *p*-value is derived using a Likelihood Ratio Test, and a subset of inferred GLM parameters can be analyzed. The resulting p-values and bootstrap confidence intervals can help assess the significance of each term in the model to gene gain and loss (Tonkin-Hill et al., 2023).

### 2.8 Egg-NOG-mapper vs. InterProScan

In this benchmarking analysis, we compared the annotation performance of EggNOG-mapper and InterProScan for *M. marinum*, focusing on metrics such as GO term accuracy, average GO terms per protein, and proteome coverage (Huerta-Cepas et al., 2017). Annotation files generated by each tool were parsed using the pandas library in Python v3.8, extracting relevant data such as GO terms and Pfam domains. The data were structured into a standardized format, capturing sets of GO terms, functional categories, and proteome coverage for both tools. To evaluate the performance of each tool, key metrics were calculated. These included the proportions of true positive (TP) and false positive (FP) annotations, which were used to assess the accuracy of GO term predictions. Additionally, the average number of GO terms per protein was computed to reflect the specificity of the annotations. Finally, proteome coverage was evaluated based on the proportion of proteins that were annotated with only TP terms, a mix of TP and FP terms, or no TP terms. This analysis provides a comparative framework for assessing annotation quality, highlighting the strengths and limitations of each tool’s functional annotation capabilities for genomic studies.

## 3 Results

### 3.1 Genomic features and ANI

**FIGURE 1:**
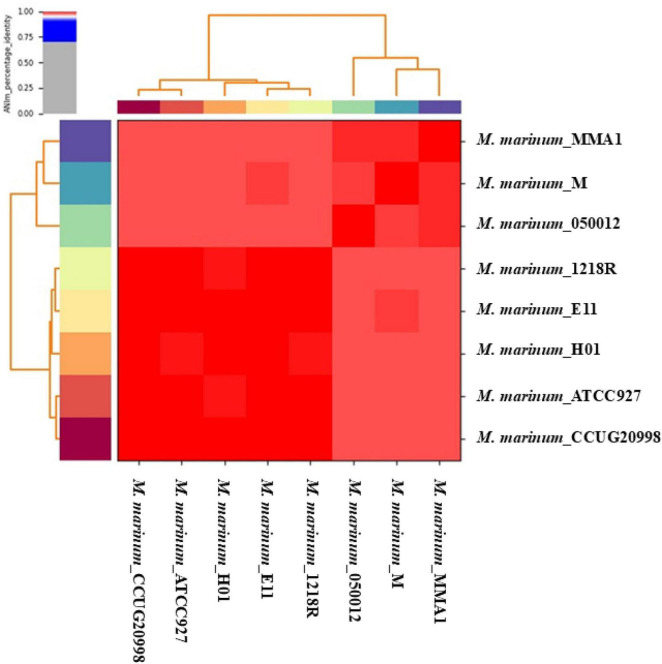
The pairwise average nucleotide identity (ANI) values for the eight complete *M. marinum* genomes are represented in this heat map, with distinct strains differentiated by color coding along the *x*- and *y*-axes. High similarity, indicated by the color red, suggests that the organisms belong to the same species.

The genomic features are represented in Supplementary Table 1, which lists the assembly accessions, organism infraspecific names, assembly release dates, number of scaffolds, and isolated species for eight complete genomes of *M. marinum*. Genetic variability between the fish isolates, such as E11, CCUG20998, and 1218R, and the human isolates, including H01, MMA1, and *Mycobacterium*, is minimal according to the ANI analysis (Figure 1). The heatmap shows that the ANI percentages are consistently high, nearing or above 98%, indicating close genetic relatedness between strains from fish and human sources. This suggests that despite the different host origins, these *M. marinum* strains share significant genomic similarity.

### 3.2 Pangenome and core genome

Pangenome analysis of *M. marinum* based on the eight complete genome sequences reveals a large proportion of core genes and significant genomic diversity. The gene presence-absence matrix (Supplementary Figure 1) showed a clear clustering of strains, with the core genome represented by 4634 gene clusters (Supplementary Figure 2). This suggests that a substantial fraction of genes is conserved across all strains, indicating evolutionary stability in essential functions. Additionally, the shell genome comprises 1390 gene clusters, reflecting moderate variability among strains. No soft-core genes were detected, while 1758 gene clusters belong to the cloud genome, representing strain-specific genes. These cloud genes highlight the presence of unique, possibly adaptive, traits linked to the strain’s environmental or host-specific pressures.

**FIGURE 2:**
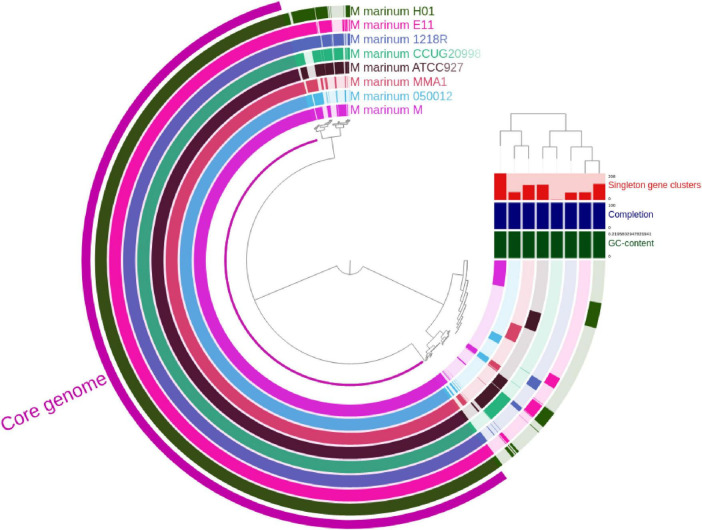
The visualization of the pangenome of *M. marinum* was conducted by performing Anvio, which incorporated a total of eight complete genomes. The dendrogram shows the phylogenetic relationship between the strains. The bars on the right of the dendrogram represent the number of singleton genes in each strain. The bar graph below the dendrogram shows the GC content of each strain.

Figure 2 displays a circular representation of the core genome and pan-genome of *M. marinum*. The outermost circle represents the core genome, which is shared by all strains. The inner circles represent the pan-genome, which consists of genes that are present in at least one strain. The pangenome analysis reveals that the core genome of *M. marinum* is relatively small, suggesting that the species has a high degree of genetic diversity. The presence of many singleton genes in each strain suggests that *M. marinum* is rapidly evolving and adapting to different environments.

**FIGURE 3:**
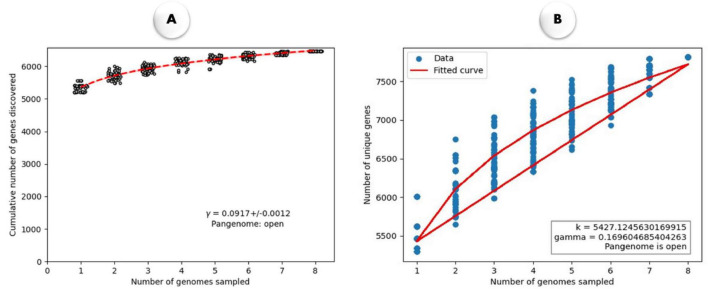
**(A)** The rarefaction curve illustrates the quantity of newly identified genes resulting from random additions to a single genome. The power-law fit equation is represented by γ = 0.0917 ± 0.0012. **(B)** Heap’s Law plot illustrates the relationship between the number of genomes sampled and the number of unique genes identified in *M. marinum*. The fitted curve (red line) follows Heap’s Law, with parameters k = 5427.124.76 and γ = 0.169, indicating an open pangenome, where the number of unique genes increases as more genomes are sampled.

Figure 3 illustrates the open nature of the pangenome using different fitting models. A power-law fit to depict the cumulative number of genes discovered as more genomes are sampled (Figure 3A). The steady slope in the fitted curve indicates continuous gene discovery, confirming that the pangenome remains open, as suggested by the equation y = 0.0917x + 0.0012y. Heap’s law (Figure 3B) shows the number of unique genes discovered with increasing genome samples and further reinforces the open pangenome model, as new unique genes continue to emerge with additional genome sampling.

### 3.3 Genome synteny analysis and biomarker identification

The Mauve software was utilized to create a visual representation of gene synteny across the genomic sequences of various *M. marinum* strains, allowing for the identification of potential genetic rearrangements based on sequence similarity (Supplementary Figure 3). The presence of gene blocks facilitated the detection of synteny, enabling the accurate identification of these regions. A comparative analysis of the genomes revealed several fragmented areas, characterized by multiple inversions and deletions. Notably, despite these disruptions, a high degree of uniformity was observed, with large, conserved segments exhibiting inversions and deletions when compared across the genomes.

### 3.4 Encoding genomic features

**FIGURE 4:**
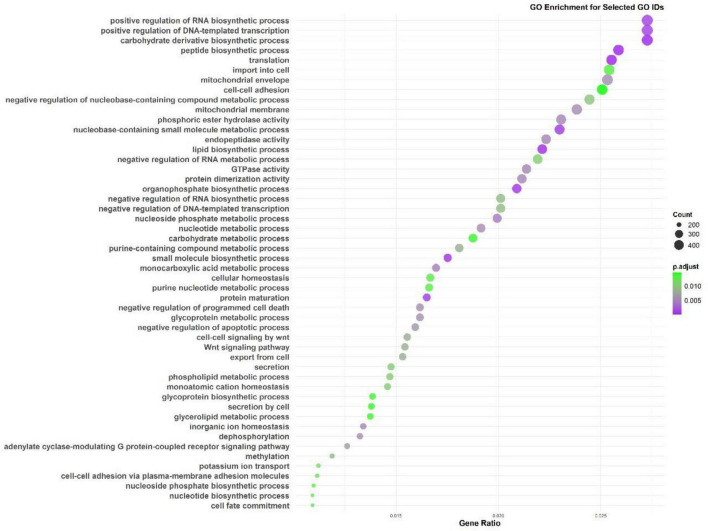
Gene Ontology (GO) enrichment analysis from the *M. marinum* pangenome annotation showing key biological processes.

**FIGURE 5:**
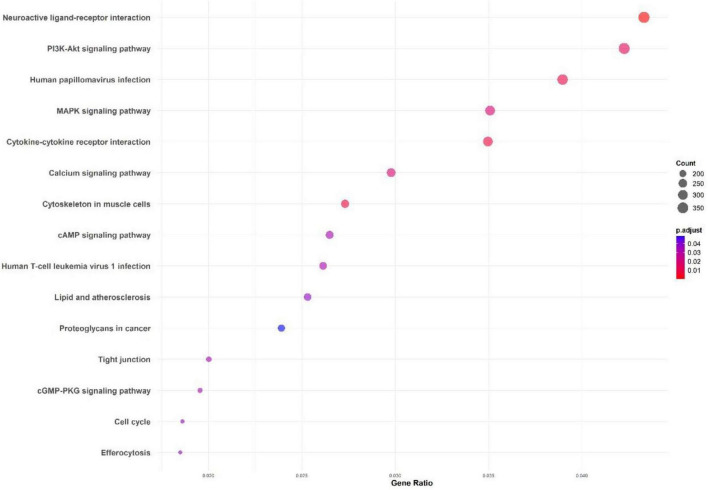
KEGG pathway enrichment analysis for the *M. marinum* pangenome annotation using eggNOG and InterProScan.

The Gene Ontology (GO) enrichment (Figure 4) and KEGG pathway analysis (Figure 5) of the *M. marinum* pangenome annotation, conducted through eggNOG and InterProScan, provide a comprehensive overview of the functional capabilities of the genes identified. The GO enrichment analysis reveals that biological processes like positive regulation of RNA biosynthetic processes, DNA-templated transcription, carbohydrate derivative biosynthesis, mitochondrial function, and cell adhesion are highly significant. These processes underscore key roles in cellular organization, metabolic activity, biosynthesis, and cell signaling. The negative regulation of nucleobase-containing compound metabolism and RNA metabolic processes also suggests intricate regulatory mechanisms at play in gene expression and metabolic control.

The KEGG pathway analysis highlights critical pathways including the PI3K-Akt and MAPK signaling pathways, both essential for cell survival, growth, and immune responses. Other enriched pathways like neuroactive ligand-receptor interaction, cytokine-cytokine receptor interaction, and calcium signaling pathways point to significant communication and signaling mechanisms that might play a role in host-pathogen interactions.

The virulence factor analysis of all the complete genomes identified several key virulence genes, highlighting important duplications: *esxB, fbpC, fbpB, esxG, esxH, eccA3, eccC3, esxM, esxA, ideR, relA, hbhA, phoP*, and *mbtH* (Table 1). Notably, the duplication of *fbpB* and *fbpC*, both associated with mycolyl transferase activity, suggests functional redundancy in adherence mechanisms. Additionally, the presence of multiple components of the Type VII secretion system, including *esxB, esxG, esxH, eccA3*, and *eccC3*, underscores the significance of this system in the virulence of *M. marinum.*

**TABLE 1:** Functional category of virulence genes in *M. marinum* pangenome.

Gene functional category	Virulence gene	Product
Effector delivery system	*esxB*	10 kDa culture filtrate antigen EsxB (CFP-10)
Adherence	*fbpC*	Secreted antigen 85-C FbpC (85C) (antigen 85 complex C) (AG58C) (mycolyl transferase 85C) (fibronectin-binding protein C)
Effector delivery system	*eccA3*	Type VII secretion system protein EccA3
Effector delivery system	*eccC3*	Type VII secretion system protein EccC3
Effector delivery system	*PE5*	PE family protein
Effector delivery system	*esxG*	Type VII secretion system protein EsxG
Effector delivery system	*esxH*	Type VII secretion system protein ESXH (10 kDa antigen) (CFP-7) (protein TB10.4)
Others	*icl*	Isocitrate lyase Icl (isocitrase) (isocitratase)
Adherence	*hbhA*	iron-regulated heparin binding hemagglutinin hbhA (adhesin)
Regulation	*ideR*	Iron-dependent repressor and activator IdeR
Regulation	*relA*	Probable GTP pyrophosphokinase RelA (ATP: GTP 3’-pyrophosphotransferase) (PPGPP synthetase I) ((P)PPGPP synthetase) (GTP diphosphokinase)
Effector delivery system	*esxM*	type VII secretion system ESX-5 protein EsxJ
Effector delivery system	*esxN*	ESX-5 type VII secretion system EsxA (ESAT-6) homolog
Effector delivery system	*Rv1794*	ESX-5 locus protein
Effector delivery system	*eccD5*	ESX-5 type VII secretion system EccD5
Effector delivery system	*mycP5*	ESX-5 type VII secretion system subtilisin-like serine protease MycP5
Effector delivery system	*eccA5*	ESX-5 type VII secretion system AAA + ATPase EccA5
Adherence	*fbpB*	Secreted antigen 85-B FbpB (85B) (antigen 85 complex B) (mycolyl transferase 85B) (fibronectin-binding protein B) (extracellular alpha-antigen)
Nutritional/Metabolic factor	*mbtH*	MbtH-like protein
Regulation	*phoP*	Possible two component system response transcriptional positive regulator PhoP
Effector delivery system	*eccA1*	ESX-1 type VII secretion system AAA + ATPase EccA1
Effector delivery system	*eccCa1*	ESX-1 type VII secretion system FtsK/SpoIIIE family protein EccCa1
Effector delivery system	*eccCb1*	ESX-1 type VII secretion system FtsK/SpoIIIE family protein EccCb1
Effector delivery system	*esxA*	6 kDa early secretory antigenic target EsxA (ESAT-6)

### 3.5 Genomic plasticity identification

The RGP analysis of the pangenome of *M. marinum* revealed significant genomic diversity among the different strains. A total of 34 RGPs were identified in the study, with some RGPs present in multiple strains, indicating conservation among different strains. Notably, antimicrobial resistance genes, such as those conferring resistance to dibekacin, netilmicin, tobramycin, and Fosfomycin, were widespread among the strains. Additionally, antibiotic-resistant murA transferase, aminosalicylate resistant, and virulence factor genes were found to be co-localized in the same RGP, suggesting potential co-regulation. The analysis also identified several modules (or genes) that were present in multiple strains, including modules 26, 32, 28, 30, and 44. Strain-specific RGPs were also observed, with strain CCUG20998 having a unique RGP (RGP_0) and strain MMA1 having a unique RGP (RGP_14). Overall, the RGP analysis highlights the complex genomic landscape of *M. marinum* and the potential for co-regulation of antimicrobial resistance and virulence genes.

### 3.6 Gene gain-loss evaluation

**FIGURE 6:**
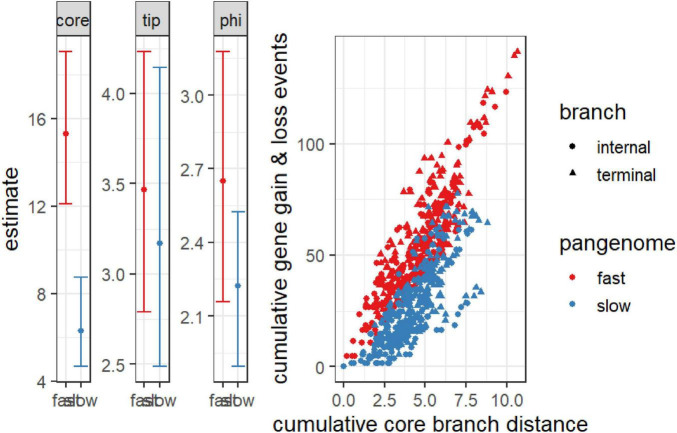
Cumulative gene gain and loss events versus cumulative core branch distance for fast and slow-evolving pangenomes. The scatter plot distinguishes between internal branches (circles) and terminal branches (triangles), with “fast” pangenomes shown in red and “slow” pangenomes in blue. The steeper accumulation of gene gain and loss events in fast-evolving pangenomes, particularly along terminal branches, indicates an open pangenome with ongoing gene acquisition over time. The accompanying bar plots display the model estimates for the core, tip, and phi terms, with confidence intervals for both fast and slow pangenomes. The significant temporal signal in the core term suggests continuous gene flow in the open pangenome, whereas the non-significant depth and dispersion parameters reflect consistent gene exchange across the phylogeny.

The analysis of the open pangenome model reveals critical insights into the temporal dynamics of gene gain and loss events. The significant negative estimate of the “core” term (–0.705, *p* = 1.02e-4), coupled with the narrow bootstrap confidence interval (–1.15, –0.255), indicates strong evidence for an open pangenome (Figure 6). This suggests that gene gain and loss events are continuously accumulating over time, characteristic of an open system where new genes are incorporated as the genome evolves. The accumulation is not temporally limited, signifying ongoing horizontal gene transfer or gene acquisition from various sources, possibly through mobile genetic elements, which are typically associated with open pangenomes. Furthermore, the non-significant estimates for “depth” (estimate = –0.0195, *p* = 4.62e-1) and “tip” (estimate = –0.153, *p* = 4.31e-1) align with the expected pattern for an open pangenome. These results suggest that there are no substantial differences in detecting older gene exchange events or gene presence at the tips, reinforcing the idea that gene gain and loss occur throughout the evolutionary history of the genomes, not confined to specific time points. The non-significance of the dispersion term also implies that the rate and size of gene exchange events remain consistent, rather than varying dramatically between different branches of the phylogeny. The plot comparing cumulative gene gain and loss events against core branch distance shows distinct patterns for fast and slow-evolving pangenomes. The rapid accumulation of genes in the fast-evolving pangenome supports the interpretation of an open system, where more gene exchange events occur over shorter evolutionary timescales, especially along terminal branches, as indicated by the denser clustering (Figure 6). This continuous acquisition of genes aligns with the expected behavior of an open pangenome, where there is no saturation point for gene gain, and new genes continue to enter the genome pool over time.

### 3.7 Benchmarking annotation data

**FIGURE 7:**
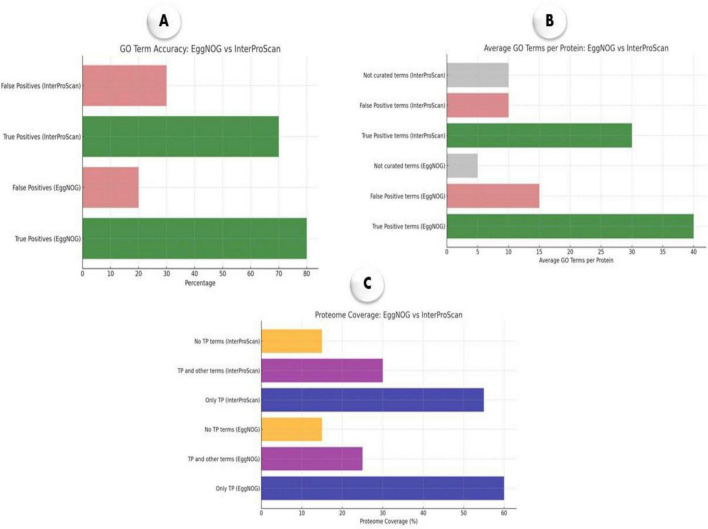
Benchmarking of EggNOG-mapper and InterProScan annotation tools for *M. marinum*. **(A)** GO term accuracy showing proportions of true and false positives; **(B)** average GO terms per protein, categorized into curated terms; **(C)** proteome coverage indicating the percentage of proteins annotated with only true positives, a mix of true and false positives, or no true positives.

The benchmarking analysis presented in Figure 7 provides a comparative evaluation of EggNOG-mapper and InterProScan for annotating *M. marinum* genomes. Figure 7A showing a higher proportion of true positive annotations for both tools, with EggNOG-mapper achieving 80% true positives compared to 70% for InterProScan. False positive rates are comparatively lower, indicating that both tools provide a reliable level of annotation accuracy, though EggNOG-mapper has an advantage. The average GO terms per protein, reveals that EggNOG-mapper assigns a higher average of curated (true positive) terms per protein, reflecting greater annotation specificity and comprehensiveness (Figure 7B). InterProScan, while also providing curated terms, shows a slightly lower average, particularly in false positive and non-curated terms, which suggests a more conservative annotation approach. Figure 7C demonstrating that both techniques offer substantial coverage exclusively with true positive (TP) annotations. EggNOG-mapper covers 60% of the proteome with TP terms only, whereas InterProScan covers 55%, indicating robust proteome-level annotation by both tools, though EggNOG-mapper marginally outperforms in exclusive TP coverage. Together, these results indicate that EggNOG-mapper provides a more extensive annotation profile across metrics, making it slightly more effective in annotation accuracy, specificity, and proteome coverage.

## 4 Discussion

*Mycobacterium marinum* presents a zoonotic threat, causing granulomatous skin lesions and deeper tissue infections in humans (Petrini, 2006). Transmission typically occurs via contact with contaminated water (Tsai et al., 2007) or injuries from fish spines (Jernigan and Farr, 2000), presenting clinically as ulcers, nodular lymphangitis, or − more severely − tenosynovitis, arthritis, or osteomyelitis (Wongworawat et al., 2003). The disease spectrum ranges from self-limiting infections to chronic, treatment-resistant cases (Aubry et al., 2002), highlighting the need to characterize both conserved and emerging virulence factors and antibiotic resistance mechanisms.

This study provides significant insights into the genomic landscape and evolutionary dynamics of *M. marinum*, a pathogen capable of infecting both fish and humans. The consistently high ANI values (≥ 98%) among strains from both hosts indicate a close genetic relationship, despite differences in host origin. These findings challenge previous observations by Das et al. (2018), likely due to our use of complete genome sequences for comparative analysis, which offers a more precise and thorough understanding of an organism’s genetic composition than analyses based on contigs, scaffolds, or chromosomes (Vasudevan et al., 2020). Our data suggest that *M. marinum* conserved survival and virulence mechanisms in diverse hosts, potentially making it as a generalist pathogen. The maintenance of such high genetic similarity across species further implies a strong evolutionary conservation of core biological functions, a trait shared by other pathogenic mycobacteria such as *M. tuberculosis* (Fisher, 2021). Moreover, the open nature of the *M. marinum* pangenome—demonstrated by Heap’s law and power-law fitting—supports its high genomic plasticity. The gradual slope of the fitted curve indicates that the discovery of new genes is ongoing and will likely continue as more genomes are analyzed. This open pangenome model stands in contrasts to closed pangenomes, such as that of *Bacillus anthracis* (Selvaraj et al., 2021), where little to no new gene acquisition occurs. Additionally, the high number of strain-specific singleton genes in *M. marinum* further highlights its rapid genome evolution, likely driven by HGT (Dumas et al., 2016) and adaptation to diverse environmental stresses. These unique genes may confer strain-specific advantages, such as enhanced resistance to environmental pathogens (e.g., *Pseudomonas aeruginosa*), where strain-specific genes are linked to niche adaptation and virulence (Jurado-Martín et al., 2021).

This study presents the first benchmarking analysis of the *M. marinum* pangenome, validating the utility of pangenomic bioinformatic tools (van Dongen and Abreu-Goodger, 2012). The significant negative estimate of the “core” term (–0.705, *p*-value = 1.02e-4) with a narrow bootstrap confidence interval (–1.15 to –0.255) confirms the presence of a robust open pangenome (Tokuda and Shintani, 2024). The non-significant estimates for “depth” (–0.0195, *p*-value = 0.462) and “istip” (–0.153, *p* = 0.431) further support the notion of continuous gene acquisition and loss throughout the pathogen’s evolutionary history. Analysis of cumulative gene gain/loss events across core branch distances shows distinct patterns, differentiating fast- from slow-evolving pangenomes. The rapid accumulation of genes in fast-evolving pangenomes supports the interpretation of an open system, where increased gene exchange occurs over shorter evolutionary timescales. Furthermore, benchmarking annotation tools revealed that EggNOG-mapper’s superiority in providing more comprehensive and accurate functional annotations for *M. marinum*, possibly attributable to its enormous annotation library and advanced prediction methods. While InterProScan remains precise, its conservative annotation approach results in fewer annotations, which may be preferred in studies prioritizing specificity.

Our comprehensive genomic analysis also highlighted critical virulence factors, particularly the *ESX1* secretion system. Similar to the findings reported in previous studies, all analyzed *M. marinum* strains exhibited partial duplication of the *ESX1* cluster, resulting in multiple copies of key virulence-associated genes, including *esxA* and *esxB* (Gao et al., 2004; Stinear et al., 2008). While *esxA* was conserved across all strains, truncated variants of *esxB* were common, indicating that *esxB* may be dispensable, whereas *esxA* appears crucial for virulence, in line with earlier reports (Rogall et al., 1990). Moreover, our study identified the existence of an additional *ESX* region − designated *ESX6* − including *esxG, esxH, esxM*, and *esxN*. The presence of these paralogs suggests compensatory mechanisms for virulence via gene conversion or epitope variation, as previously proposed (Derbyshire and Gray, 2014; Uplekar et al., 2011). This study is the first to report the presence of virulence-associated genes (e.g., *mycP5, relA, fbpB, mbtH, phoP*, and *fbpC*) in *M. marinum*. These genes are involved in effector delivery, stress response, nutrient acquisition, and host adherence, and likely contribute significantly to the pathogenic potential of *M. marinum*. The identification of gene duplications and the presence of paralogs suggest a complex evolutionary strategy aimed at maximizing adaptability and virulence. These insights not only reinforce previous studies but also contribute novel data regarding the virulence strategies employed by *M. marinum*, emphasizing the importance of *ESX* gene clusters in mycobacterial pathogenesis. While the current study sheds light on the potential of SNPs as genetic markers for pathogenicity and antibiotic resistance, future investigation should utilize high-fidelity sequencing platforms and orthogonal techniques. This is especially crucial given the variability in whole genome sequencing quality (e.g., depth, coverage, and error rates) associated with various technologies that may affect the accuracy of SNPs detection.

The analysis of RGP revealed extensive genomic diversity, identifying 34 RGPs, with some conserved across multiple strains. This marks the first detailed characterization of RGPs in *M. marinum* and highlights their role in environmental adaptation. Notably, antimicrobial resistance genes (e.g., dibekacin, netilmicin, tobramycin, and fosfomycin) were found co-localized with virulence genes such as *esxB and esxA*, suggesting possible co-regulation driven by HGT. Strain-specific RGPs, particularly in CCUG20998 and MMA1, reflect the pathogen’s capacity for genomic adaptability of *M. marinum*. The co-localization of resistance and virulence determinants presents promising targets for therapeutic strategies (Beceiro et al., 2013; Lazar et al., 2023) and enhances our understanding of *M. marinum*’s resilience in various environments.

In conclusion, this study demonstrates significant genomic and evolutionary insights with potential applications in diagnostic and aquaculture biosecurity. The identification of strain-specific SNPs in conserved genes provides a basis for developing rapid molecular diagnostics, including targeted PCR assays or CRISPR-based assays (Li et al., 2024). Similarly, conserved virulence genes could serve as biomarkers for multiplex qPCR aimed at detecting pathogenic strains in aquaculture systems (Chirakos et al., 2020; Gcebe et al., 2018). Furthermore, the co-occurrence of antibiotic resistance and virulence genes within RGPs highlights the urgent need for integrated surveillance strategies in both clinical and aquaculture settings. Future validation of these markers under field conditions will be critical to translating these findings into practical applications.

## 5 Conclusion

This study offers valuable genomic insights into *Mycobacterium marinum*, shedding light on its adaptability, virulence mechanisms, and potential as a generalist zoonotic pathogen. The high ANI values across strains, the open pangenome structure, and the abundance of singleton genes indicate ongoing evolution driven by gene acquisition and host adaptation. The identification of critical virulence factors, such as *ESX*1, *ESX6*, and novel genes like *mycP5, relA*, and *fbpC*, along with co-localized resistance genes in regions of RGPs, underscores the pathogen’s genomic plasticity. These findings contribute to our understanding of *M. marinum* pathogenesis and play an important role for improved diagnostics, surveillance, and therapeutic strategies.

## Data Availability

The datasets analyzed during the current study are available in the National Center for Biotechnology Information (NCBI) (https://www.ncbi.nlm.nih.gov). Details of the data retrieved from the NCBI database are included in Supplementary Table 1.

## Footnotes

1
https://github.com/SethCommichaux/Heap_Law_for_Roary

2
https://github.com/pinbo/msa2snp

3
https://github.com/tseemann/abricate

4
https://github.com/labgem/PPanGGOLiN

5
https://github.com/gtonkinhill/panstripe
