# Supplementary material for: Benchmarking pangenome dynamics and horizontal gene transfer in Mycobacterium marinum evolution
Source: Front Microbiol. 2025 Jun 17;16:1537826. doi: 10.3389/fmicb.2025.1537826 (PMC12209367; doi:10.3389/fmicb.2025.1537826)
Supplement: Supplementary file 1 [file Supplementary_file_1.docx]

**Supplementary Figures**


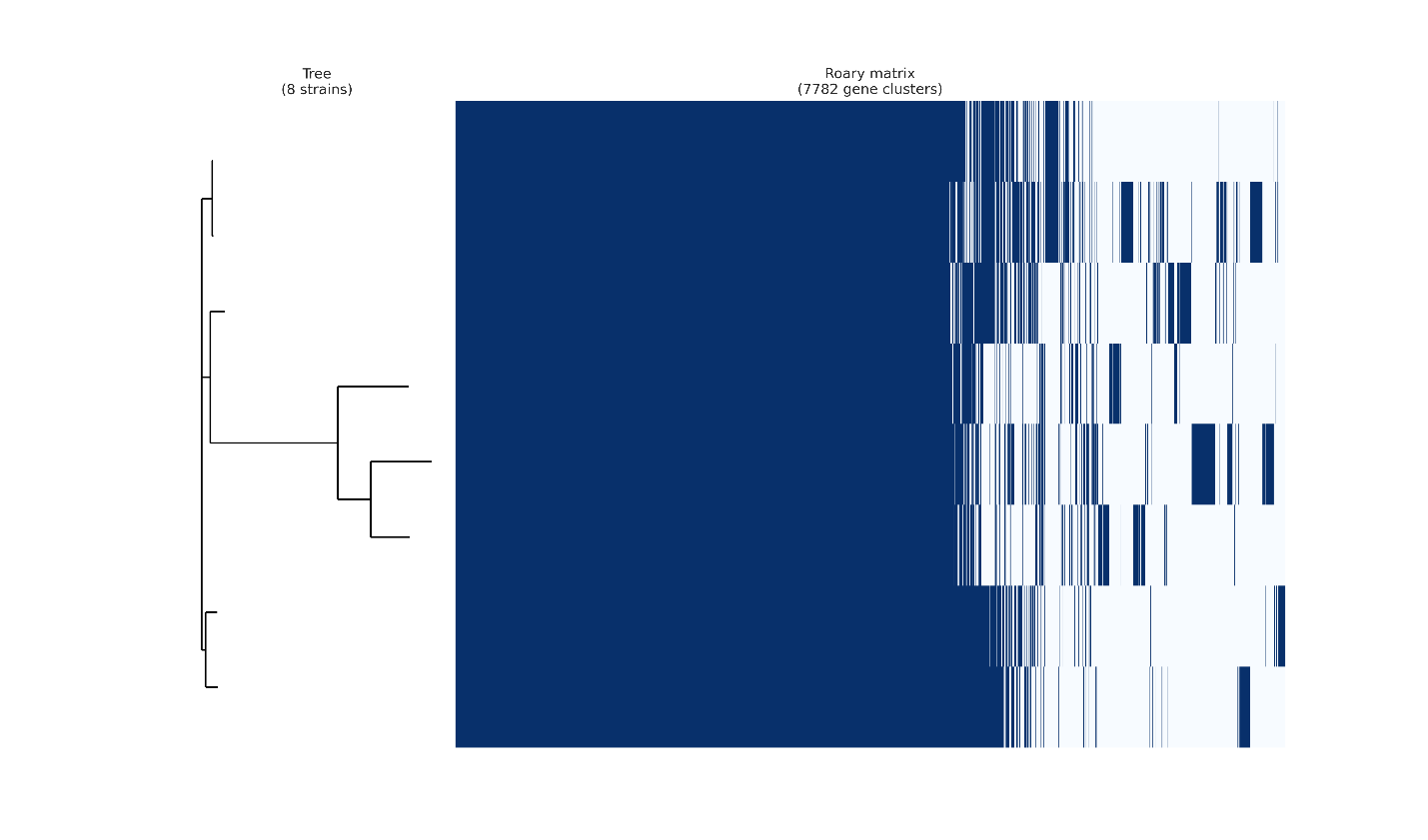


**Figure S1.** Phylogenetic tree and gene presence-absence matrix of eight Mycobacterium marinum strains (GCA_000723425.2, GCA_003391395.1, GCA_003391415.1, GCA_028583325.1, GCA_028451225.2, GCA_016745295.1, GCA_000018345.1, and GCA_003609695.1). The phylogenetic tree shows the evolutionary relationships among the strains, while the corresponding gene presence-absence matrix indicates the distribution of 7782 gene clusters across the genomes.


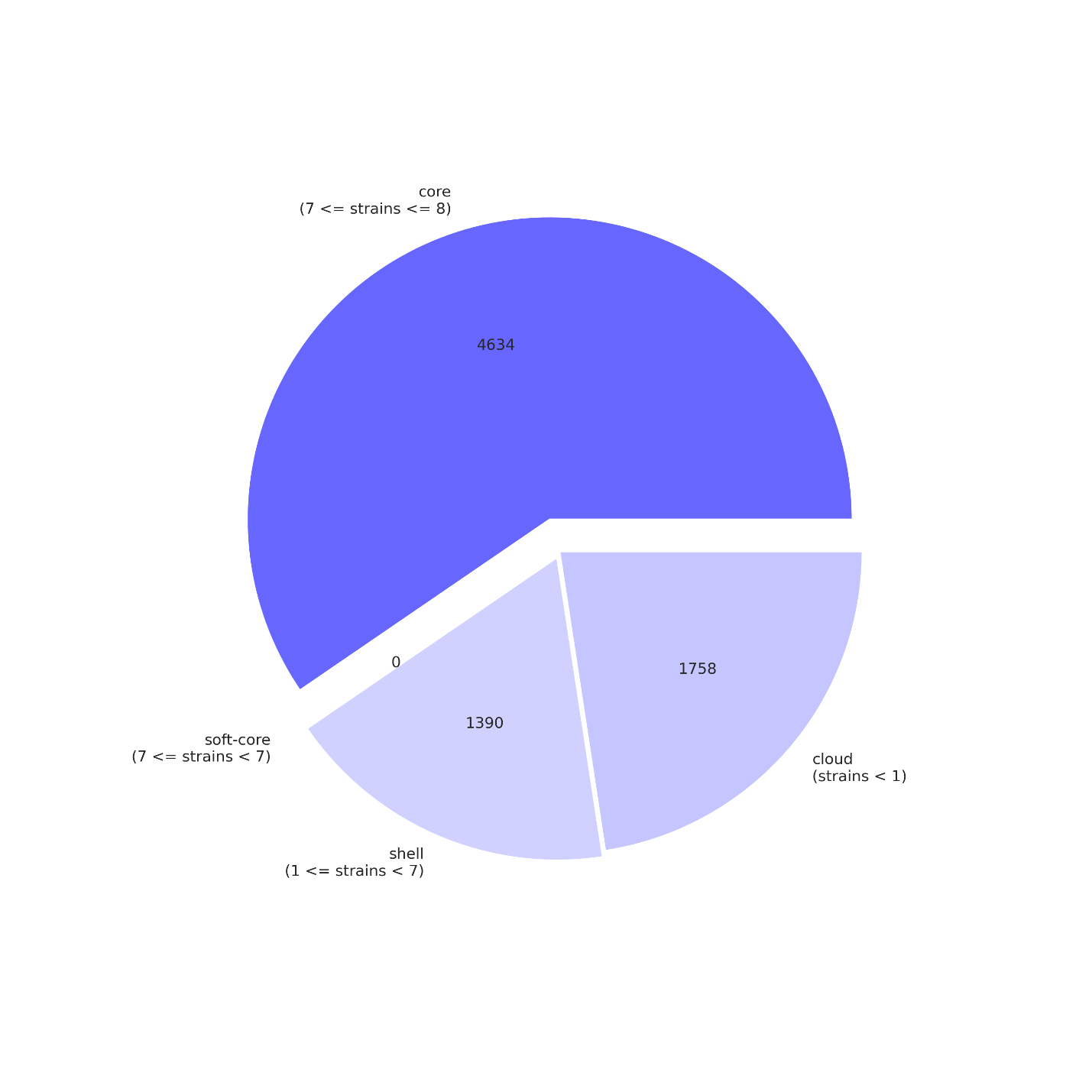


**Figure S2.** Pangenome composition of eight *Mycobacterium marinum* genomes (GCA_000723425.2, GCA_003391395.1, GCA_003391415.1, GCA_028583325.1, GCA_028451225.2, GCA_016745295.1, GCA_000018345.1, and GCA_003609695.1). The pangenome is divided into core genes (4634 gene clusters), shell genes (1390 gene clusters), and cloud genes (1758 gene clusters).


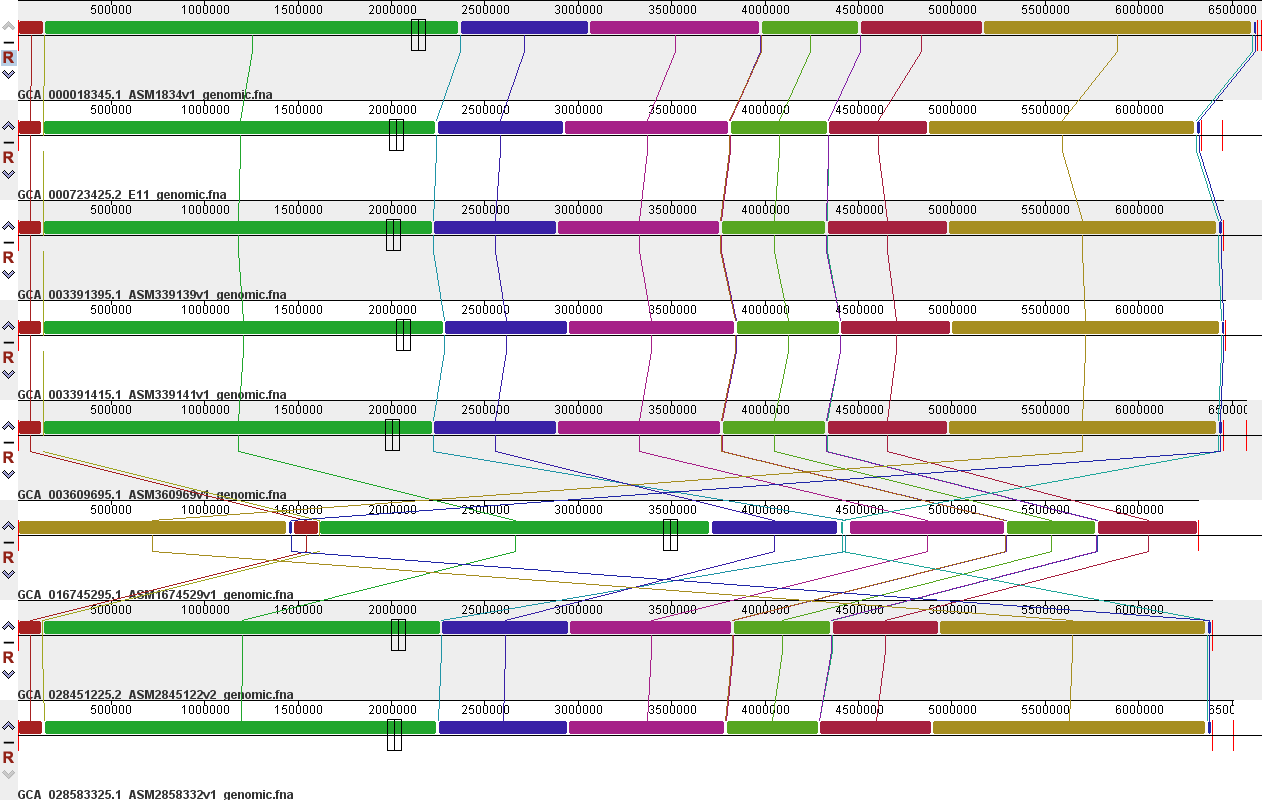


**Figure S3**. Genome synteny analysis. The genomes were broken down into contiguous fragments, which were then displayed. Each fragment is represented by a distinct shade, indicating a specific region that has undergone significant evolutionary changes, including inversions, translocations, or deletions.
