# Supplementary material for: Benchmarking pangenome dynamics and horizontal gene transfer in Mycobacterium marinum evolution
Source: Front Microbiol. 2025 Jun 17;16:1537826. doi: 10.3389/fmicb.2025.1537826 (PMC12209367; doi:10.3389/fmicb.2025.1537826)
Supplement: Supplementary file 2 [file Supplementary_file_2.docx]

**Supplementary Tables**

**Table S1.** Details of all the complete genomes of *M. marinum* used for the analysis in this study.

| **Assembly Accession** | **Genome Size (Bp)** | **Organism Infraspecific Names Strain** | **Assembly Release Date** | **Assembly Stats Number of Scaffolds** | **Isolated Species** |
| --- | --- | --- | --- | --- | --- |
| GCA_000723425.2 | 6,336,293 | E11 | 7/2/2015 | 2 | Fish |
| GCA_003391395.1 | 6,453,310 | CCUG20998 | 8/15/2018 | 1 | Fish |
| GCA_003391415.1 | 6,467,35 | 1218R | 8/15/2018 | 1 | Fish |
| GCA_028583325.1 | 6,393,732 | H01 | 2/10/2023 | 2 | Fish |
| GCA_028451225.2 | 6,393,703 | 050012 | 3/10/2023 | 1 | Human |
| GCA_016745295.1 | 6,321,947 | MMA1 | 1/22/2021 | 1 | Human |
| GCA_000018345.1 | 6,636,827 | M | 4/15/2008 | 2 | Human |
| GCA_003609695.1 | 6,451,936 | ATCC 927 | 2/7/2018 | 2 | Fish |

**Table 2.** Differential genes and their characteristic SNPs in the *M. marinum* genomes.

| **Genes** | **Annotation** | **Position, SNPs unique for *Mycobacterium marinum*** |
| --- | --- | --- |
| *gyrA* | DNA gyrase subunit A | 173 A > C/G, 356 A > C/T, 480 A > C/G,  627 G > A/C, 642 G > C/T,  837 G > C/T, 1162 A > C/G,  1215 T > C/G, 1278 G > C/T,  1404 T > C/G, 1415 T > A/C,  1471 A > C/T, 1677 A > C/G,  1941 T > A/G, 1968 C > G/T, |
| *ftsW* | putative lipid II flippase FtsW | 51 T > C/G, 99 T > A/G,  267 C > A/G, 651 A > C/T,  699 G > C/A, 761 T > C/G,  787 G > C/A, 852 C > A/G,  915 C > A/G, 984 C > A/G,  1245 C > A/G, 1314 A > C/G, |
| dnaK | chaperone protein | 195 T > -/C  858 G > T/C  930 C > T/G  1010 A > G/C  1101 T > G/C  1224 G > T/-  1242 A > G/C  1311 G > A/C  1323 T > G/C  1681 G > T/C |
| *recF* | DNA replication and repair protein RecF | 453 T > G/C,  1026 C > G/T,  1029 G > A/C, |
